# ﻿A new genus of soft coral (Octocorallia, Malacalcyonacea, Cladiellidae) and three new species from Indo-Pacific coral reefs

**DOI:** 10.3897/zookeys.1188.110617

**Published:** 2024-01-10

**Authors:** Catherine S. McFadden, Yehuda Benayahu, Kaveh Samimi-Namin

**Affiliations:** 1 Department of Biology, Harvey Mudd College, Claremont, CA 91711, USA Harvey Mudd College Claremont United States of America; 2 School of Zoology, George S. Wise Faculty of Life Sciences, Tel Aviv University, Ramat Aviv, 69978, Tel Aviv, Israel Tel Aviv University Tel Aviv Israel; 3 Marine Evolution and Ecology Group, Naturalis Biodiversity Center, P.O. Box 9517, 2300 RA Leiden, Netherlands Marine Evolution and Ecology Group, Naturalis Biodiversity Center Leiden Netherlands; 4 Department of Biology, University of Oxford, Oxfordshire, Oxford OX1 3SZ, UK University of Oxford Oxford United Kingdom; 5 Natural History Museum, Cromwell Road, London SW7 5BD, UK Natural History Museum London United Kingdom

**Keywords:** DNA barcoding, molecular phylogeny, new combination, northern Red Sea, *Ofwegenum* gen. nov., Oman, Réunion, sclerites, target-enrichment, taxonomy, ultraconserved elements

## Abstract

Molecular systematic studies of the anthozoan class Octocorallia have revealed widespread incongruence between phylogenetic relationships and taxonomic classification at all levels of the Linnean hierarchy. Among the soft coral taxa in order Malacalcyonacea, the family Alcyoniidae and its type genus *Alcyonium* have both been recognised to be highly polyphyletic. A recent family-level revision of Octocorallia established a number of new families for genera formerly considered to belong to Alcyoniidae, but revision of *Alcyonium* is not yet complete. Previous molecular studies have supported the placement of *Alcyoniumverseveldti* (Benayahu, 1982) in family Cladiellidae rather than Alcyoniidae, phylogenetically distinct from the other three genera in that family. Here we describe a new genus, *Ofwegenum***gen. nov.** to accommodate *O.verseveldti***comb. nov.** and three new species of that genus, *O.coronalucis***sp. nov.**, *O.kloogi***sp. nov.**, and *O.colli***sp. nov.**, bringing the total number of species in this genus to four. *Ofwegenum***gen. nov.** is a rarely encountered genus so far known from only a few locations spanning the Indian and western Pacific Oceans. We present the morphological characters of each species and use molecular data from both DNA barcoding and target-enrichment of conserved elements to explore species boundaries and phylogenetic relationships within the genus.

## ﻿Introduction

Zooxanthellate soft corals belonging to the octocorallian order Malacalcyonacea are among the most common, conspicuous, and ecologically important sessile organisms on shallow-water coral reefs throughout the Indo-Pacific; on some reefs, total percent cover of soft corals may exceed that of the reef-building scleractinian corals (Tursch and Tursch 1982; Dinesen 1983; Dai 1991; Fabricius 1997; Fabricius and Dommisse 2000). Despite their ubiquity, the taxonomy of even the most common genera of soft corals is poorly understood, and until recently a majority of the large, fleshy, zooxanthellate genera that dominate space on shallow reefs were classified in family Alcyoniidae Lamouroux, 1812 (Fabricius and Alderslade 2001). This family, along with its type genus *Alcyonium* Linnaeus, 1758, has long been a repository for genera and species whose morphological characters do not cleanly fit the diagnoses of other families (Alderslade 2000; Williams 2000; McFadden and van Ofwegen 2013). A recent revision of class Octocorallia based on novel phylogenomic evidence has now re-circumscribed Alcyoniidae to include only azooxanthellate and mostly cold-water taxa (McFadden et al. 2022). New families have been established and previously suppressed families reinstated to accommodate the tropical genera formerly considered to be alcyoniids (McFadden et al. 2022), and some species of *Alcyonium* have been transferred to new genera and families (Alderslade 2000; Williams 2000; McFadden and Hochberg 2003; McFadden and van Ofwegen 2013, 2017). Revision of *Alcyonium* is, however, far from complete, and among the species still classified in that genus is *A.verseveldti* (Benayahu, 1982), originally described as *Metalcyoniumverseveldti*, a rare species known only from a few collections in the Red Sea. Molecular phylogenetic studies suggest that this species belongs to family Cladiellidae McFadden, van Ofwegen & Quattrini, 2022, but not to any of the established genera within that family (i.e., *Cladiella* Gray, 1869, *Klyxum* Alderslade, 2000, and *Aldersladum* Benayahu & McFadden, 2011; see Benayahu et al. 2012; McFadden et al. 2022).

Pfeffer (1889) established the genus *Metalcyonium* (Octocorallia, Alcyoniidae) for two species of soft corals from South Georgia, *M.clavatum* Pfeffer, 1888 and *M.capitatum* Pfeffer, 1888, without designating a type species. His description of this genus lacked much detail, merely noting that the colonies were unbranched and club-shaped (i.e., clavate) with a distinct polyp-bearing region (polyparium) and a narrower, sterile stalk. He also observed that the polyps retracted into calyces that were distributed over the surface of the polyparium (Pfeffer 1889). He described the sclerites as warty “Doppelspindeln” (a term often used for a spindle with a median waist; Bayer et al. 1983), denser in the calyces than in the stalk, and absent from the neck of the polyp. Kükenthal (1906) concluded that in all aspects of its morphology other than the unbranched colony growth form *Metalcyonium* resembled *Alcyonium*. He relegated *Metalcyonium* to the status of a subgenus, diagnosing it succinctly as “Alcyonien von unverzweigter, walzenförmiger oder konischer Körperform” (Kükenthal 1906: 43, i.e., alcyonians with unbranched, cylindrical, or conical colony form). Subsequent authors (e.g., Thomson 1910, 1921) did not accept Kükenthal’s revision and assigned additional species of soft corals with unbranched, clavate or capitate colony forms to *Metalcyonium* throughout the early 20^th^ century.

Utinomi (1958) further validated the genus, stating “it is undoubted that *Metalcyonium* is a unique group embracing the species which are clavate, capitate or mushroom-shaped and ordinarily unbranched in form” (1958: 110). He suggested, however, that *M.clavatum*, which he erroneously stated to be the type species of *Metalcyonium*, might belong instead to the genus *Bellonella* Gray, 1862 because its colony shape is relatively digitiform rather than capitate. In a subsequent publication, Utinomi (1964) designated *M.capitatum* as the type species of *Metalcyonium*. Williams (1986) argued that the capitate colony growth form alone did not justify the separation of the genus *Metalcyonium* from *Alcyonium* because species such as *M.patagonicum* May, 1899 and *M.variabile* J. S. Thomson, 1921 can exhibit a range of forms intermediate between digitiform and capitate. He transferred all capitate species of *Metalcyonium*, including the type species *M.capitatum*, to *Alcyonium*, thereby invalidating the genus. Verseveldt and Bayer (1988) then redescribed Pfeffer’s original type material and moved both *M.clavatum* and *M.capitatum* to *Bellonella*, synonymising *Metalcyonium* with that genus.

Among the species of *Metalcyonium* transferred by Williams (1986) to *Alcyonium* was *M.verseveldti* Benayahu, 1982, found in the warm tropical waters of the northern Red Sea. Molecular phylogenetic analyses that have included this species place it in a clade with the tropical Indo-Pacific genera *Cladiella* Gray, 1869 and *Klyxum* Alderslade, 2000 (Benayahu et al. 2012), phylogenetically distant from *Alcyonium* (see McFadden et al. 2022).

**Figure 1.**
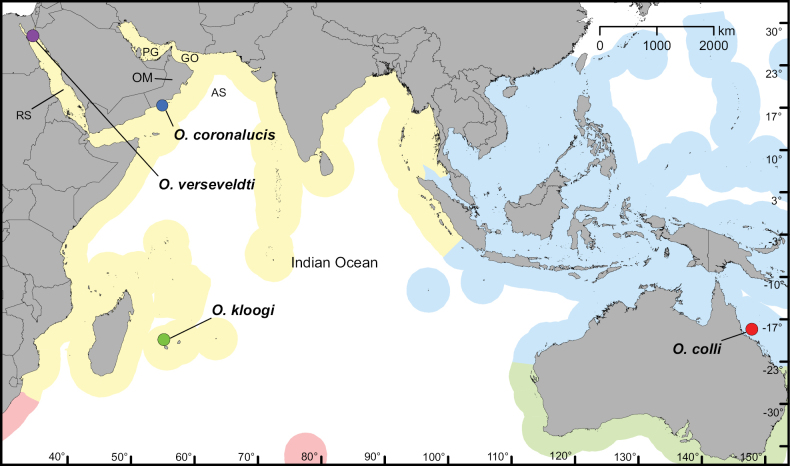
Distribution of the *Ofwegenum* gen. nov. species in the Indo-Pacific region. The colour shades represent the different marine realms. Yellow = West Indo-Pacific, blue = Central Indo-Pacific, red = East Africa, green = temperate Australasia; PG = Persian Gulf, AS = Arabian Sea, RS = Red Sea, GO = Gulf of Oman, OM = Oman.

Here, we re-examine the type material and establish a new genus for *M.verseveldti*. In addition, we describe three new species of the genus from the Indian and western Pacific Oceans (Fig. 1). We present features of the sclerites of each species and examine the genetic distinctions among species using single-locus DNA barcodes and multi-locus sequence data from target-enrichment of conserved elements (UCEs and exons).

## ﻿Materials and methods

### ﻿Morphological studies

The study examined the holotype and paratypes of *Metalcyoniumverseveldti* Benayahu, 1982 and other relevant material deposited at the museums listed below. Morphological features, including shape and dimensions of the preserved colonies, were recorded; terminology follows McFadden et al. (2022) and Bayer et al. (1983). To examine the sclerites, tissue samples were treated with 10% sodium hypochlorite followed by repeated rinses in distilled water. Wet preparations of the clean sclerites were examined under a Nikon Eclipse 80i light microscope at ×100–200 magnification. Scanning Electron Microscope (SEM) mounts were prepared from the sclerites. The mounts were coated with Pd/Au or Cr and viewed under a Quanta 200 FEG (Field Emission Gun) ESEM operated at 5–20 kV or Au coated and viewed under a Hitachi TM-1000 ESEM at Tel Aviv University and Jeol 6480LV SEM operated at 10 kV, with Pt coating at Naturalis Biodiversity Center, Leiden.

### ﻿Abbreviations

**NBC**Naturalis Biodiversity Center (formerly Rijksmuseum van Natuurlijke Historie, RMNH) Leiden, The Netherlands;

**NTM**Museum and Art Gallery of the Northern Territory, Darwin, Australia;

**QM**Queensland Museum, Brisbane, Australia;

**SMNHTAU**Steinhardt Museum of Natural History at Tel Aviv University, Tel Aviv, Israel;

**UF**Florida Natural History Museum, Florida, United States.

### ﻿Molecular phylogenetic analyses

DNA was extracted from EtOH-preserved tissue samples using a DNeasy Blood & Tissue Kit (Qiagen, Inc.). Fragments of the mitochondrial *mtMutS* and *COI* (+*igr1*) genes and nuclear *28S rDNA* were amplified by polymerase chain reaction (PCR) and sequenced using published primers and protocols (McFadden et al. 2014). New sequences were added to an alignment of family Cladiellidae analysed previously by Benayahu et al. (2012) (Table 1) that included one of the specimens described here and realigned using the L-INS-i method in MAFFT (Katoh et al. 2005). Pairwise genetic distances (uncorrected p) among taxa for each gene region were determined using MEGA v.5 (Tamura et al. 2011).

**Table 1.** GenBank accession numbers for specimens of *Ofwegenum* gen. nov. and other genera of Cladiellidae included in molecular analyses (Fig. 16). Raw UCE sequence reads are deposited under project number PRJNA1035147.

Species	Museum	Locality	*mtMutS*	*28S*	*COI*	UCEs
*Ofwegenumcoronalucis*	UF 15819	Oman	NA	OR483157	OR487130	NA
	SMNHTAU_Co_39048	Oman	OR487121	OR483155	OR487131	SAMN 38083212
	UF 17263	Oman	OR487122	OR483156	OR487134	SAMN 38083211
	UF 15877	Oman	OR487123	OR483158	OR487132	NA
	BOMAN-09174	Oman	OR487124	OR483159	NA	NA
	UF 15882	Oman	OR487125	OR483160	OR487133	NA
Ofwegenumaff.coronalucis	SMNHTAU_ Co_38223	Aquarium trade, USA	OR487121	OR483157	OR487130	SAMN 38083213
*Ofwegenumverseveldti*	SMNHTAU_ Co_33097	Israel	GU356012	JX991219	GU355978	SAMN 38083214
*Ofwegenumkloogi*	SMNHTAU_ Co_34426	Reunion	OR487117	OR483152	OR487128	SAMN 38083210
	SMNHTAU_ Co_38229	Reunion	OR487118	OR483153	NA	NA
*Ofwegenumcolli*	NTM C13089	Australia	OR487120	NA	NA	NA
*Aldersladumjengi*	SMNHTAU_ Co_33607	Taiwan	JX991144	JX991201	JX991220	NA
*Aldersladumsodwanum*	SMNHTAU_ Co_31520	Kenya	JX991193	JX991213	JX991236	NA
*Cladiellaaustralis*	SMNHTAU_ Co_36313	Taiwan	MH516863	MH516878	MH516513	SAMN 38083203
	SMNHTAU_ CO_36912	Taiwan	MH516570	MH516881	MH516515	SAMN 38083204
	SMNHTAU_ Co_36987	Taiwan	MH516571	MH516882	MH516516	SAMN 38083205
	SMNHTAU_ Co_36042	Madagascar	OR487126	OR483164	OR487135	SAMN 38083206
*Cladiellabottae*	SMNHTAU_ Co_34648	Taiwan	JX991145	JX991204	JX991223	NA
*Cladiellakashmani*	SMNHTAU_ Co_32334	Kenya	JX991195	JX991215	JX991238	NA
	SMNHTAU_ Co_32246	Kenya	JX991194	JX991214	JX991237	NA
*Cladiellapachyclados*	SMNHTAU_ Co_33604	Taiwan	JX991146	JX991206	JX991225	NA
	SMNHTAU_ Co_35507	Palau	JX991197	JX991216	JX991240	NA
*Cladiellasphaerophora*	SMNHTAU_ Co_34132	Israel	GQ342471	JX203653	GQ342386	NA
*Cladiellatuberculoides*	SMNHTAU_ Co_34686	Taiwan	JX991227	JX991148	JX991208	NA
	SMNHTAU_ Co_34642	Taiwan	JX991226	JX991147	JX991207	NA
*Cladiellatuberosa*	SMNHTAU_ Co_34669	Taiwan	JX991149	JX991209	JX991228	NA
*Klyxum* sp.	UF 2684	N. Marianas	OR487127	OR483162	NA	SAMN 38083207
	QM G330915	Australia	NA	OR483163	NA	SAMN 38083208
	CKT396	Taiwan	NA	OR483161	NA	SAMN 38083209
*Klyxumadii*	SMNHTAU_ Co_32636	Kenya	JX991199	JX991217	JX991242	NA
*Klyxumflaccidum*	SMNHTAU_ Co_32221	Kenya	JX991200	JX991218	JX991243	NA
*Klyxumutinomii*	SMNHTAU_ Co_34639	Taiwan	JX991151	JX991212	JX991232	NA
	SMNHTAU_ Co_34127	Israel	GQ342476	JX203654	GQ342392	NA

Preliminary phylogenetic analyses of each gene region using PhyML (Guindon and Gascuel 2003) revealed congruence of gene trees, therefore genes were concatenated for further analyses. To minimise the effects of missing data on the analyses, a 471 bp fragment of the *mtMutS* gene was concatenated with *28S rDNA*; *COI* was not included in the concatenated alignment. Optimal models of evolution for each gene (*mtMutS*: HKY+F; *28S*: TN+F+I) were found using ModelFinder (Kalyaanamoorthy et al. 2017) and a maximum likelihood tree was constructed using IQTree v. 2.1.2 (Minh et al. 2020) with an edge-linked partition model (Chernomor et al. 2016) and 10,000 ultrafast bootstraps (Hoang et al. 2018). A partitioned analysis was run using MrBayes v. 3.2.1 (Ronquist et al. 2012), applying a HKY model to *mtMutS* and a GTR+ G model to *28S rDNA*. MrBayes was run for 3,000,000 generations (until standard deviation of split partitions < 0.01) with a burn-in of 25% and default Metropolis coupling parameters.

### ﻿Target-enrichment sequencing of conserved elements

For one or a few representatives of each species and several outgroup taxa (*Cladiella*, *Klyxum*), DNA was quantified using a Qubit 2.0 fluorometer and quality-checked (for 260:230 and 260:280 ratios) using a NanoDrop spectrophotometer. DNA samples (300–1000 ng) were sent to Arbor Biosystems (Ann Arbor, MI) for library preparation, target enrichment and sequencing. Libraries were prepared using a Kapa Hyper Prep Kit (Kapa Biosystems) with dual-indexed iTru adaptors. myBaits protocol v. 4 (Arbor Biosystems) was used to target and enrich pools of 8 libraries using the octocoral-v. 2 bait set of Erickson et al. (2020). Enriched libraries were sequenced on one lane of Illumina HiSeq 2500 (150 bp PE reads).

Sequences were processed using the phyluce pipeline (Faircloth 2016) as outlined in Erickson et al. (2020). Briefly, reads were cleaned using illumiprocessor (Faircloth 2013) and Trimmomatic v. 0.35 (Bolger et al. 2014), then assembled into contigs using Spades v. 3.1 (Bankevich et al. 2012) with –careful and –cov-cutoff 2 parameters. *phyluce_assembly_match_contigs_to_probes* was used to identify loci by matching probes to contigs with a minimum coverage of 70% and minimum identity of 70%. *phyluce_assembly_get_fastas_from_match_counts* was used to extract loci which were then aligned using MAFFT v. 7.130b (Katoh and Standley 2013). Sequences for seven outgroup taxa belonging to the genera *Cladiella* and *Klyxum* were included in the alignment. Aligned loci were edge-trimmed using *phyluce_align_seqcap_align*, and *phyluce_align_get_only_loci_with_min_taxa* was used to concatenate loci into a data matrix with 75% of taxa present for each locus. A maximum likelihood tree was constructed using IQTree v. 2.1.2 (Minh et al. 2020). ModelFinder (Kalyaanamoorthy et al. 2017) was used to select the best model of evolution (-m MFP), and an analysis was run with 1000 ultrafast bootstraps (Hoang et al. 2018) and 1000 replicates of an SH-like approximate likelihood ratio test (SH-aLRT) (Guindon et al. 2010).

## ﻿Results

### ﻿Systematics

**Subphylum Anthozoa Ehrenberg, 1831**

**Class Octocorallia Haeckel, 1866**

**Order Malacalcyonacea McFadden, van Ofwegen & Quattrini, 2022**

**Family Cladiellidae McFadden, van Ofwegen & Quattrini, 2022**

#### ﻿ Ofwegenum gen. nov.

Taxon classificationAnimaliaMalacalcyonaceaCladiellidae

01C59237-4328-5D46-AFCA-14DF292E1245

https://zoobank.org/10C92BD3-D724-42A5-A050-09F112AA33B7

##### Diagnosis.

Soft corals with encrusting or capitate growth forms; small (1–2 cm diameter), stalked capitula may be joined basally to form a low mat. Polyps monomorphic, non-retractile but contractile; pinnules with or without terminal branches. Coenenchymal sclerites are spindles and rods, smooth but with low, simple tubercles and areas of thickening forming concentric, raised rings. Polyp sclerites similar, usually arranged ‘en chevron’ in the polyp body, lacking a distinct collaret-and-points arrangement. Tentacles and pinnules contain numerous platelets and flattened rods (i.e., finger- biscuits, see Bayer et al. 1983) with varying features such as lateral median constrictions, side notches, or depressions at one or both ends resembling a figure-eight, arranged mostly on the aboral side of the tentacles. Some species also have tiny sclerites around the mouth. Live colonies with blue, green, or brown colouration in the coenenchyme; pinnules brown. Sclerites colourless. Zooxanthellate.

##### Type species.

*Metalcyoniumverseveldti* Benayahu, 1982: 197–201.

##### Etymology.

The generic name *Ofwegenum* (gender: neuter) honours the late Dr. Leendert P. van Ofwegen (1953–2021), a close friend and an eminent octocoral taxonomist (Hoeksema 2021), in memory of his prolific contribution to the knowledge of this group.

### ﻿Key to the species of *Ofwegenum* gen. nov.

**Table:** 

1	Colonies encrusting, not capitate and without stalk	***O.kloogi***
–	Colonies capitate, with stalk	**2**
2	Crosses and irregular sclerites up to 0.05 mm, around the polyp mouth	***O.coronalucis***
–	No sclerites around the polyp mouth	**3**
3	Coenenchymal sclerites up to 0.70 mm long, tentacle sclerites mostly figure-eight platelets	***O.verseveldti***
–	Coenenchymal sclerites up to 0.40 mm long, tentacle sclerites mostly flattened rods or bone-shaped platelets up to 0.15 mm long	***O.colli***

#### ﻿ Ofwegenum colli sp. nov.

Taxon classificationAnimaliaMalacalcyonaceaCladiellidae

81117156-4381-5608-BEEC-E86E497CD450

https://zoobank.org/E72328A2-B94F-4574-BBD2-BE72255AF6F6

Figs 1 , 3A, B , 4 , 5 , 6

##### Material examined.

***Holotype*.** Australia • Queensland, N.E. Bay Great Palm Island; 18.7500°S, 146.6500°N; 6–7 m depth; 22 April 1981; coll. J. Coll; silty bottom, on a dead coral; NTM C13089.

***Paratypes*.** Australia • 7 colonies, same data as holotype; NTM C015578 • 5 colonies, same data as holotype; NTM C3827 • 1 colony, same data as holotype; NTM C3828 • 3 colonies, same data as holotype; May 1982; NTM C3829.

##### Description.

The holotype is a fragment of a colony measuring 14 by 13 mm (Fig. 3A). Its polypary expands over a 2 mm thick, spreading crust-like base. The surface of the polypary features some grooves, and the contracted polyps, up to 1 mm in diameter, are visible as low mounds (Fig. 3A). The coenenchyme has sclerites in the form of spindles (with tapered ends) and rods (with blunt ends) up to 0.50 mm long, with low, simple tubercles or areas of thickening forming concentric, raised rings (Fig. 4A). The polyp body contains similar but shorter rods that appear to be arranged ‘en chevron’ when the polyps are extended. The size of the sclerites decreases along the polyp body towards the base of the tentacles (Fig. 4A).

The tentacles and pinnules contain numerous platelets and flattened rods (i.e., finger-biscuits, see Bayer et al. 1983) up to 0.10 mm long (Fig. 4B) arranged on the aboral side of the tentacles. Some of these sclerites have lateral median constrictions, side notches, or depressions at one or both ends resembling a figure-eight shape, and some have bulbous ends resembling bones (Fig. 4B).

##### Colour.

The ethanol-preserved colony is cream.

##### Morphological variations.

The paratype colony NTM C3829 has smoother and shorter spindles and rods compared to the holotype (0.20 vs. 0.50 mm, respectively: Figs 4A, 6A). The tentacle sclerites are up to 0.15 mm long (Fig. 6B) compared to up to 0.10 mm in the holotype (Fig. 4B). The holotype NTM C13089 has some platelets with wider ends, resembling the shape of a bone (Fig. 4B), which are not present in the other type material of this species (Figs 5B, 6B).

##### Remarks.

This species is capitate with smaller bud-like capitula occasionally emerging from the stalk. The sclerites of the paratypes correspond to those of the holotype but differ a bit in size. This species has the largest tentacle sclerites among the congeners, up to 0.15 mm long (Figs 4–6). No information is available on the living features of this species.

##### Distribution.

Queensland, Australia.

##### Etymology.

The species is named after the collector of the material, Prof. John Coll of James Cook University, North Queensland, a renowned chemical ecologist who has contributed prominently to the knowledge of soft corals.

#### ﻿ Ofwegenum coronalucis sp. nov.

Taxon classificationAnimaliaMalacalcyonaceaCladiellidae

DC7FE5C6-91EC-5217-9599-6A36E8027DE2

https://zoobank.org/1030A306-9E82-4E0E-8565-9D3D157A5406

Figs 1 , 2A, B , 3H, I , 7 , 8 , 9 , 10A–D , 11

##### Material examined.

***Holotype*.** Oman • Dhofar, Mirbat, Michel’s Reef; 16.9433°N, 54.7300°E; 25–30 m depth; 20 January 2022; coll. C.S. McFadden and K. Samimi-Namin; UF 17263 (BOMAN–08362).

***Paratype*.** Oman • same data as holotype; SMNHTAU_Co_39048 (BOMAN–08351).

##### Other material.

Oman • Dhofar, Mirbat, Frankincense; 16.9662°N, 54.6900°E; 24–30 m depth; 19 Jan 2022; coll. C.S. McFadden; UF 15819 (BOMAN–08345) • Dhofar, Mirbat, near Frankincense; 16.9688°N, 54.6877°E; 24–29 m depth; 21 Jan 2022; coll. C.S. McFadden and K. Samimi-Namin; UF15882 (BOMAN–09175) • same collection data as for preceding; UF 15877 (BOMAN–09166) • same collection data as for preceding; in situ photo, microscope slides and molecular data only; BOMAN–09174. Unknown • Aquarium trade, Chicago, IL, USA; July 2013; coll. A. Parrin; SMNHTAU_Co_38223.

##### Description.

The holotype consists of several fragments of a colony; the largest is 10 mm in diameter (Fig. 3H). The colony consists of multiple capitate polyparia on sterile stalks; side branches connect adjacent stalks to one other at the base to form an encrusting mat. Most polyps are contracted, with polyps widely set on the polyparium (Figs 3H, 9A, B).

**Figure 2.**
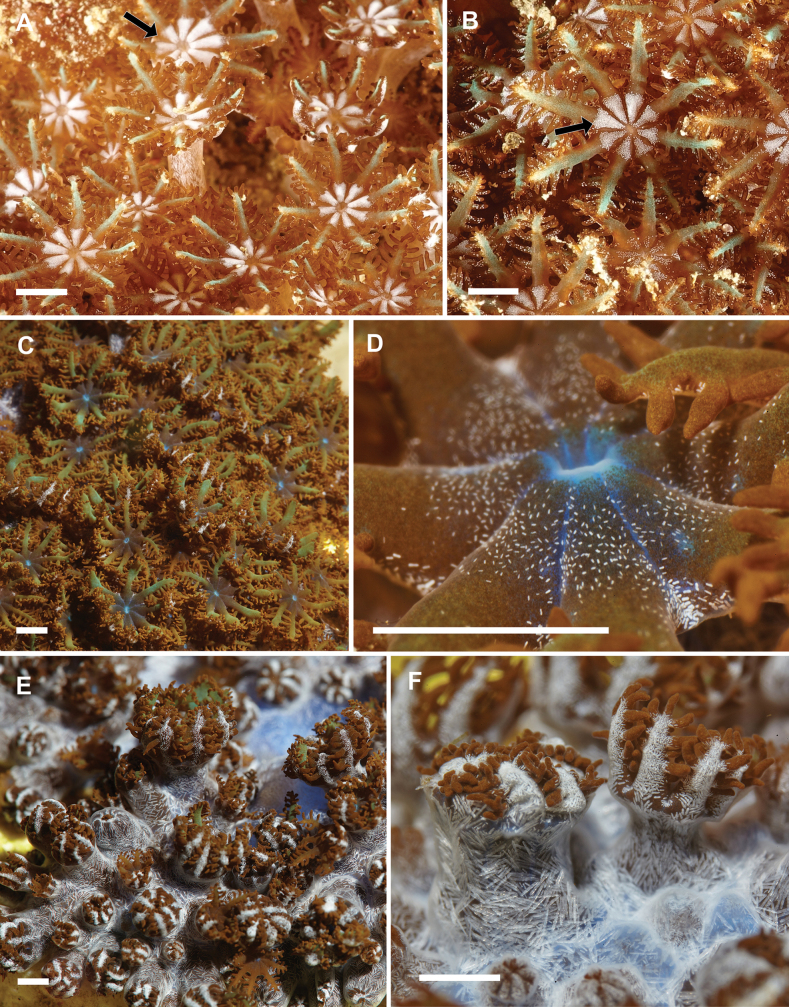
Morphological details of live *Ofwegenum* gen. nov. polyps **A, B** close up of *Ofwegenumcoronalucis* sp. nov., holotype, UF 17263; arrows indicate the concentration of minute sclerites around the mouth opening and base of the tentacles **C–F** unknown species of *Ofwegenum* gen. nov. from the aquarium trade. Scale bars: approximately 5 mm (photographs **A, B** K. Samimi-Namin **C–F** Daniel Knop).

**Figure 3.**
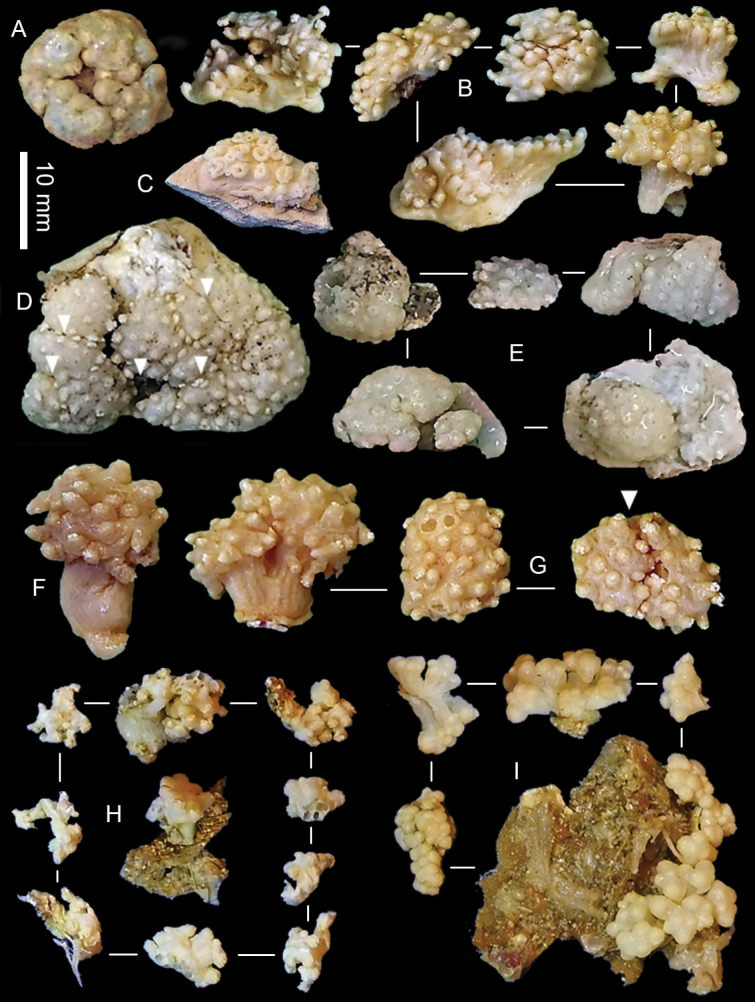
Preserved type colonies of *Ofwegenum* gen. nov. **A***O.colli* sp. nov., holotype NTM C13089 **B***O.colli* sp. nov. several paratype colonies NTM C015578 **C**O.aff.coronalucis, SMNHTAU_Co_38223 **D***O.kloogi* sp. nov. holotype SMNHTAU_Co_34426, grooves on polypary are indicated by arrows, distal ends of tentacles protrude from polyp mounds **E***O.kloogi* sp. nov., several paratype colonies SMNHTAU_Co_38299 **F***O.verseveldti* comb. nov., holotype SMNHTAU_Co_25554 **G***O.verseveldti* comb. nov., paratypes, SMNHTAU_Co_25544, grooves on polypary are indicated by arrow **H***O.coronalucis* sp. nov., holotype, UF 17263 **I***O.coronalucis* sp. nov., paratype SMNHTAU_Co_39048).

**Figure 4.**
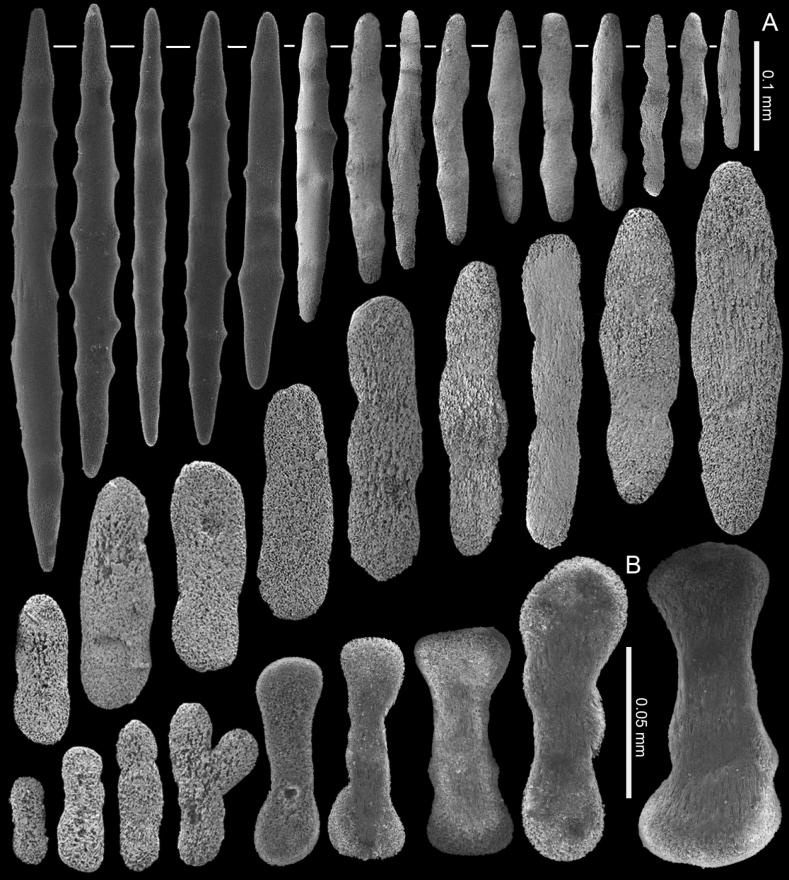
*Ofwegenumcolli* sp. nov., holotype NTM C13089 **A** sclerites of the coenenchyme and polyp body **B** sclerites of the tentacles.

**Figure 5.**
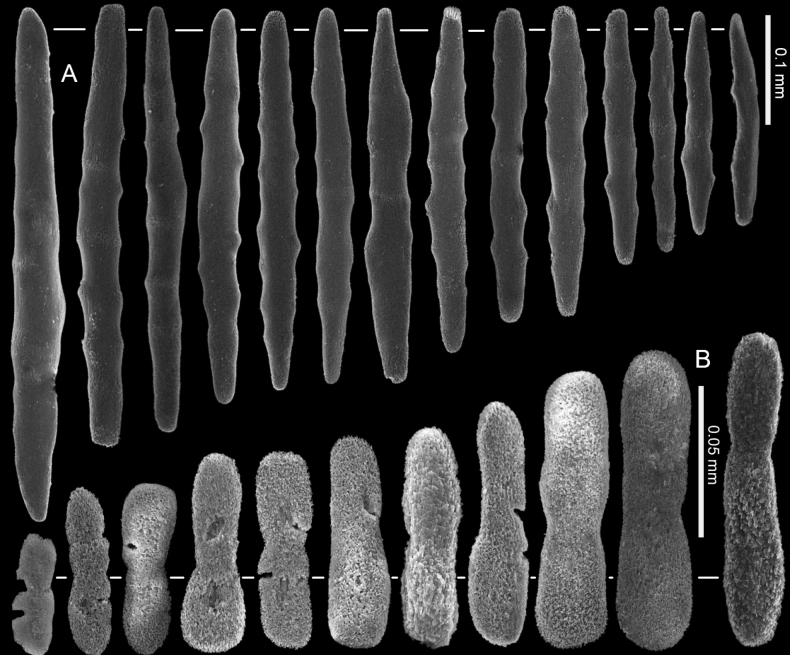
*Ofwegenumcolli* sp. nov., paratype NTM C3827 **A** sclerites of the coenenchyme and polyp body **B** sclerites of the tentacles.

**Figure 6.**
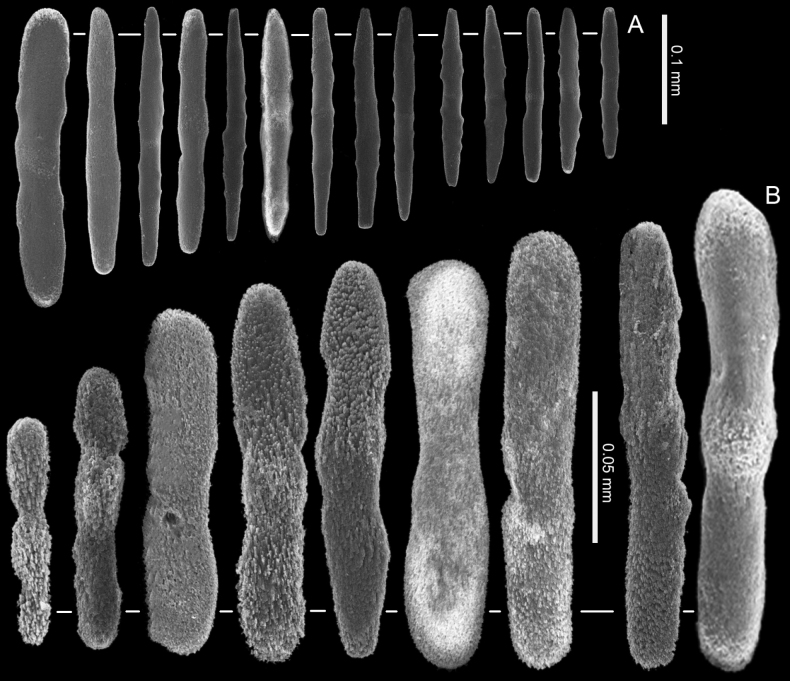
*Ofwegenumcolli* sp. nov., paratype NTM C3829 **A** sclerites of the coenenchyme and polyp body **B** sclerites of the tentacles.

**Figure 7.**
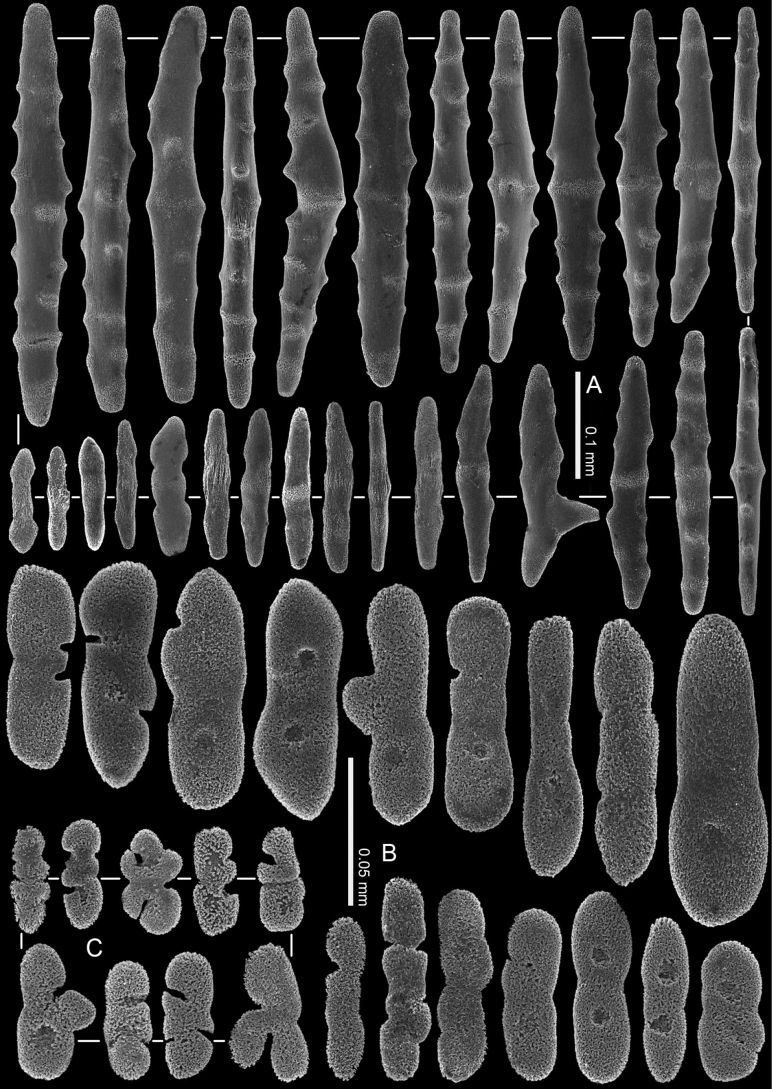
*Ofwegenumcoronalucis* sp. nov., holotype UF 17263 **A** sclerites of the coenenchyme and polyp body **B** sclerites of the tentacles **C** sclerites around the polyp mouth opening. Scale at **B** also applies to **C**.

Sclerites of the coenenchyme are spindles and rods up to 0.40 mm long with low, simple tubercles or areas of thickening forming concentric, raised rings (Fig. 7A). The polyp body contains similar but shorter rods that appear to be arranged ‘en chevron’ when the polyp is extended (Fig. 2A). These sclerites are usually blunt and have a crystalline texture at both ends (Fig. 7A). The length of the sclerites decreases along the polyp body towards the base of the tentacles (Fig. 7A).

The tentacles and pinnules contain numerous platelets and flattened rods (i.e., finger-biscuits) up to 0.10 mm long (Fig. 7B), arranged on the aboral side of the tentacles (Fig. 2A, B). Some of these sclerites have median constrictions, side notches, or depressions at one or both ends resembling figure-eight shapes (Fig. 7B). There are also numerous irregularly shaped platelets with side notches or side branches, up to 0.05 mm in length (Fig. 7C), that are distributed around the mouth and base of the tentacles on the oral side. These sclerites are reflective in light (Fig. 2A, B).

##### Colour.

In life, colonies appear brown with blue-green tentacles. After preservation in ethanol, they are creamy white. Sclerites colourless.

##### Morphological variations.

**Figure 8.**
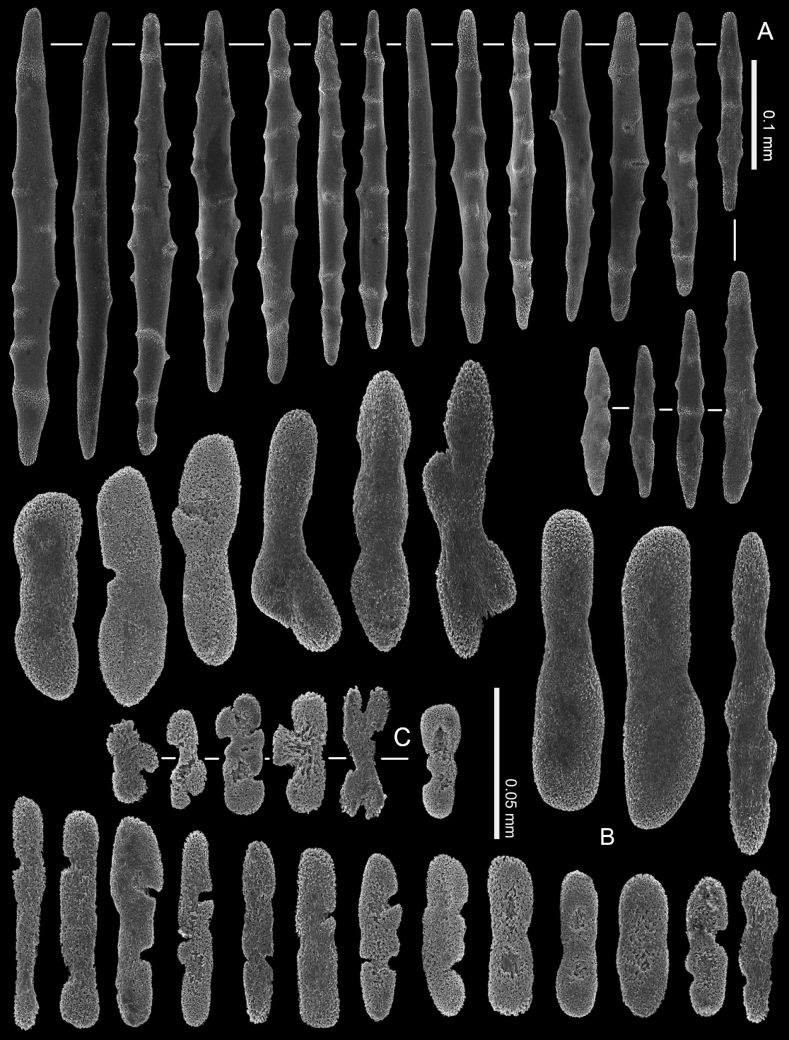
*Ofwegenumcoronalucis* sp. nov., UF 15882 **A** sclerites of the coenenchyme and polyp body **B** sclerites of the tentacles **C** Sclerites around polyp mouth opening. Scale bars: 0.05 mm (**B, C**).

**Figure 9.**
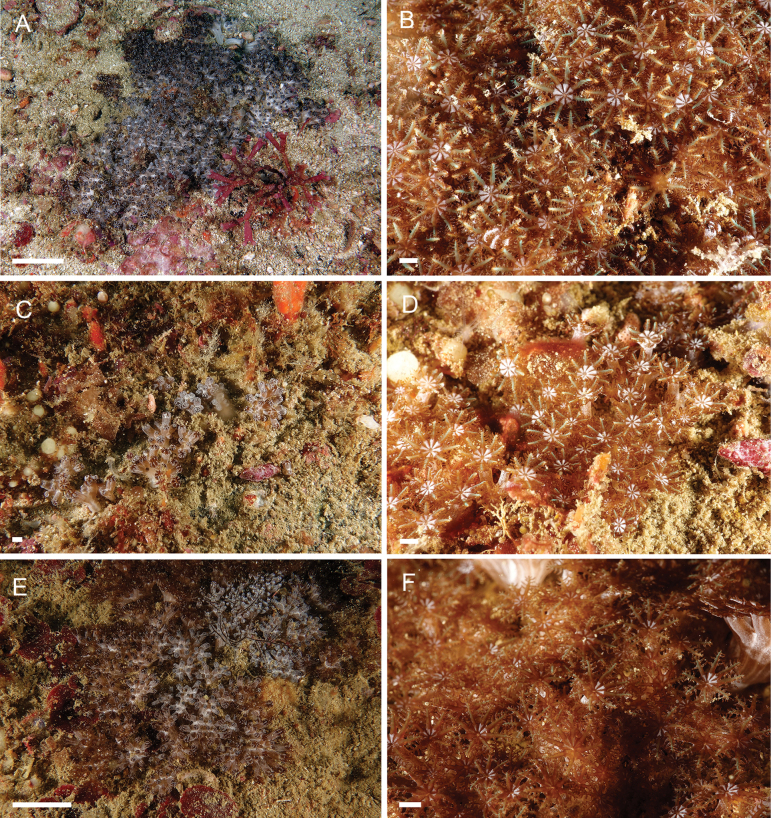
*Ofwegenumcoronalucis* sp. nov. **A, B** colony and polyps of holotype, UF 17263 **C, D** colony and polyps of paratype, SMNHTAU_Co_39048 **E, F** colony and polyps of UF 15882. Scale bars: ~ 50 mm (**A, E**); ~ 5 mm (**B–D, F**) (photographs K. Samimi-Namin).

**Figure 10.**
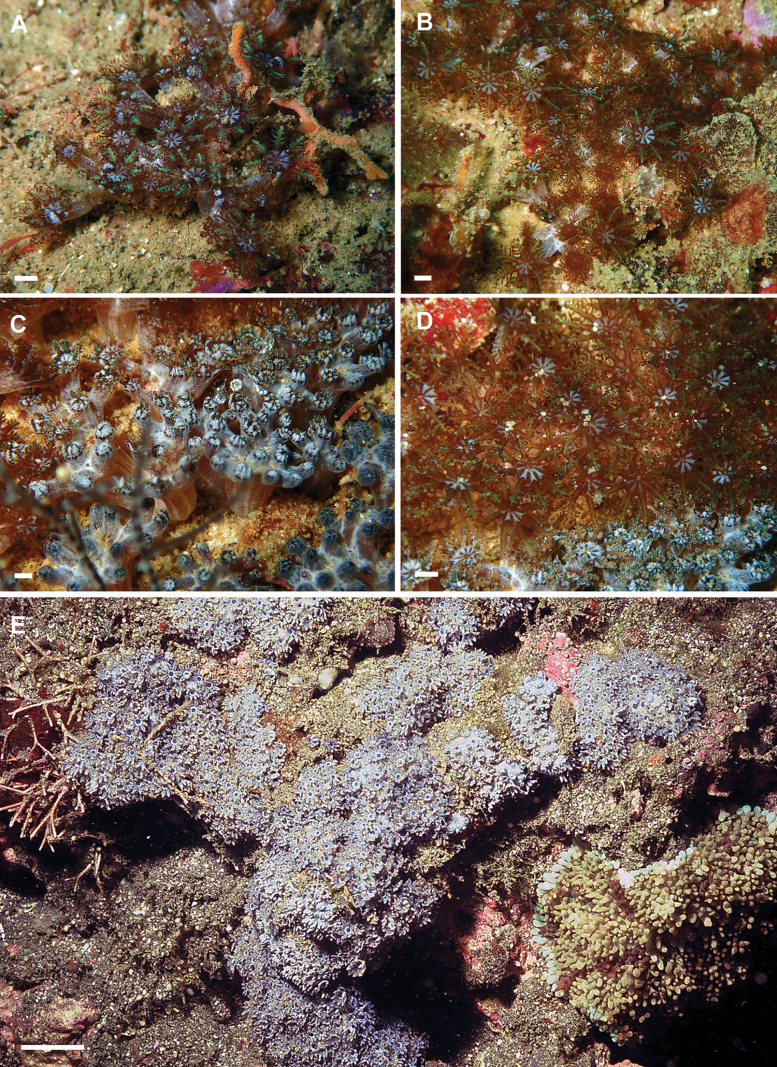
**A, B***Ofwegenumcoronalucis* sp. nov., UF 15877 **C, D***Ofwegenumcoronalucis* sp. nov., BOMAN–09174 **E***Ofwegenumkloogi* sp. nov. holotype SMNHTAU_Co_34426. (Photos **A–D** C. S. McFadden **E** Y. Benayahu). Scale bars: ~ 5 mm (**A–D**); 5 cm (**E**).

UF 15882 and BOMAN–09174 have slightly thinner spindles and rods both in the coenenchyme and polyp body (Fig. 8A). In addition, the polyp sclerites have fewer side notches and depressions compared to the holotype (Fig. 8B). Photos of the live specimens suggest that some of the polyps do not have the reflective sclerites around the mouth (Figs 9E, F, 10C, D).

**Figure 11.**
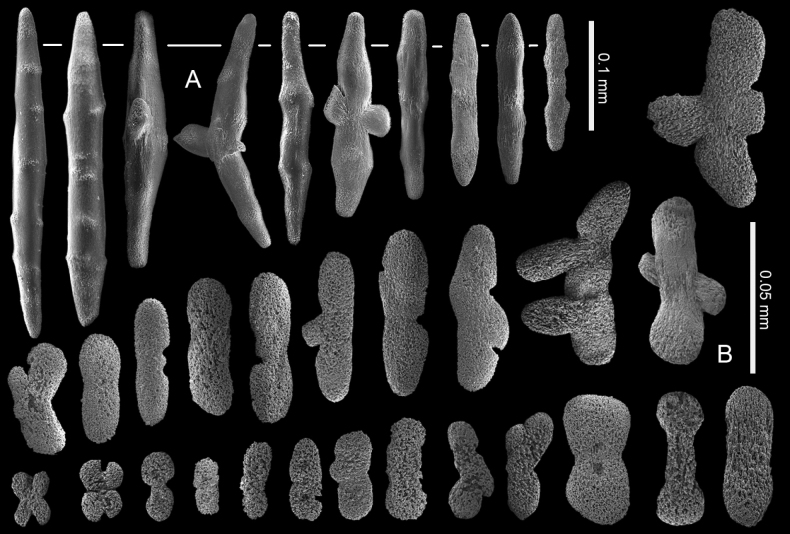
Ofwegenumaff.coronalucis SMNHTAU_Co_38223 **A** sclerites of the coenenchyme and polyp body **B** sclerites of the tentacles.

SMNHTAU_Co_38223 comes from the aquarium trade in the U.S. Its commercial source is assumed to be Jakarta, Indonesia (A. Parrin, pers. comm. 12 Aug 2013), but the original collection locality remains unknown. This colony is tentatively assigned as O.aff.coronalucis based on its sclerite features and genetic similarity to this species (Fig. 16). However, it differs from the other material in having a blue colour in the coenenchyme and shorter tentacle sclerites up to 0.07 mm long. Such differences might be due to a prolonged exposure to the artificial aquarium environment.

##### Remarks.

*Ofwegenumcoronalucis* sp. nov. differs from its congeners in having irregularly shaped sclerites with side notches or side branches around the polyp mouth that reflect light (Figs 2A, B, 7C, 8C). Additionally, the tentacle platelets have narrow median constrictions compared to the other species (Figs 7B, 8B).

##### Distribution.

Oman.

##### Etymology.

The species name is from the Latin *corona* (crown) and *lucis* (of light), referring to the reflective ring of sclerites around the polyp mouth in the live specimens.

#### ﻿ Ofwegenum kloogi sp. nov.

Taxon classificationAnimaliaMalacalcyonaceaCladiellidae

E596371B-7978-5B5A-9CF7-8393F5160177

https://zoobank.org/F1E4D927-3C21-494B-9B53-B4EA88B49817

**Figure 12.**
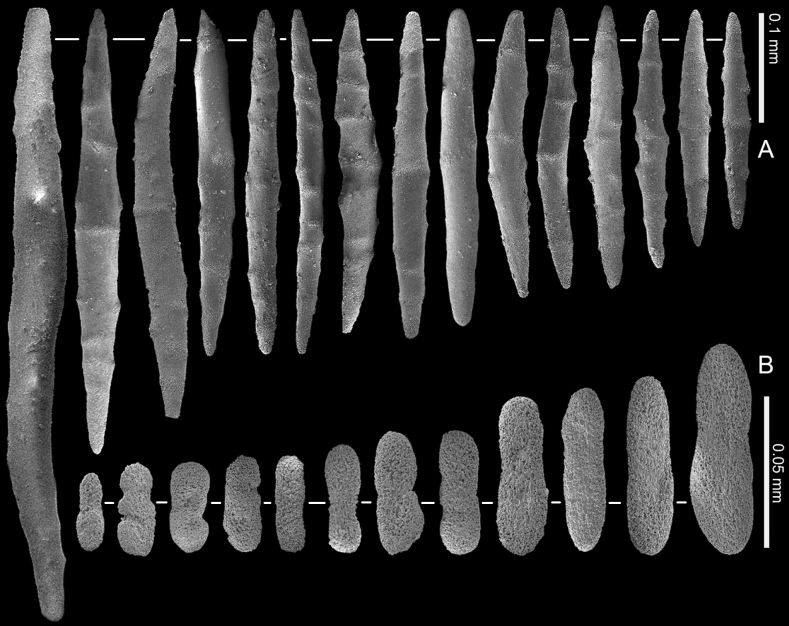
*Ofwegenumkloogi* sp. nov. holotype SMNHTAU_Co_34426 **A** sclerites of the coenenchyme and polyp body **B** tentacle sclerites, with ellipsoidal platelets and flattened rods with lateral notches.

**Figure 13.**
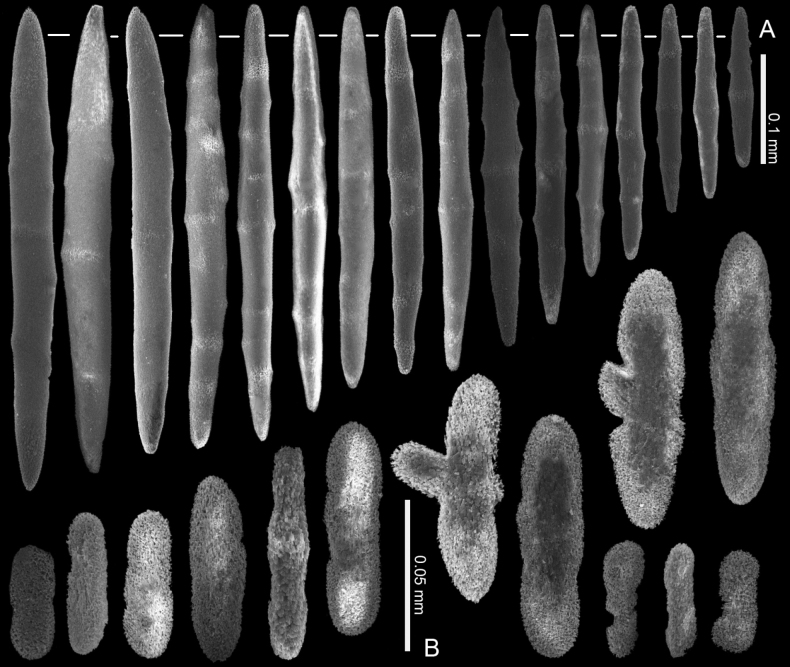
*Ofwegenumkloogi* sp. nov., paratype, SMNHTAU_Co_38229 **A** sclerites of the coenenchyme and polyp body **B** tentacle sclerites, with ellipsoidal platelets and flattened rods with lateral notches.

Figs 1 , 3D, E , 10E , 12 , 13

##### Material examined.

***Holotype*.** La Réunion • Saint-Paul, Cap la Houssaye; 21.0174°S, 55.2376°E; 17 m depth; 8 April 2008; SMNHTAU_Co_34426.

***Paratype*.** La Réunion • 13 colonies/fragments; same data as holotype; SMNHTAU_Co_38229.

##### Description.

The holotype is an encrusting colony, measuring 28 by 25 mm, attached to a calcareous fragment by a thin spreading base (<1 mm thick). The polypary features several narrow grooves (Fig. 3D). The polyps appear as low mounds. The distal tips of the tentacles occasionally protrude from the top of the polyp mounds.

The coenenchyme sclerites are spindles and rods up to 0.50 mm long, with low, simple tubercles or areas of thickening forming concentric, raised rings (Fig. 12A). The polyp body contains shorter spindles, up to 0.30 mm long (Fig. 12A), which appear to be arranged ‘en chevron’ when the polyp is extended. The length of the sclerites decreases along the polyp body towards the base of the tentacles (Fig. 12A).

The tentacles and the pinnules contain numerous platelets and flattened rods (i.e., finger-biscuits) up to 0.07 mm long (Fig. 12B), arranged on the aboral side of the tentacles. Some of these sclerites have lateral median constrictions, side notches, or depressions at one or both ends (Fig. 12B).

##### Colour.

In life the expanded tentacles are pale grey with an underlying bluish tint. The polyps have a blue mouth opening and blue line along the tentacles (Fig. 10E). The ethanol-preserved holotype is pale grey in colour.

##### Morphological variations.

Paratype SMNHTAU_Co_38229 has slightly longer tentacle sclerites and shorter coenenchymal sclerites compared to the holotype (Fig. 13).

##### Remarks.

This species features a distinct encrusting growth form and surface grooves on its polypary, most probably indicating a process of colony fission (Fig. 3D, E). Its tentacle sclerites are mainly ellipsoidal platelets and flattened rods with shallow to no median constrictions (Figs 12B, 13B). The colonies grow in dense patches on the reef (Fig. 10E).

##### Distribution.

La Réunion.

##### Etymology.

The species is named after the late Prof. Yoel Kloog, biochemist, former Dean of the Faculty of Life Sciences, Tel Aviv University, in honour of his friendship and lifetime contributions to science.

#### ﻿ Ofwegenum verseveldti

Taxon classificationAnimaliaMalacalcyonaceaCladiellidae

(Benayahu, 1982) comb. nov.

E9CBC24A-DD09-5C77-9E05-30489E7B6684

**Figure 14.**
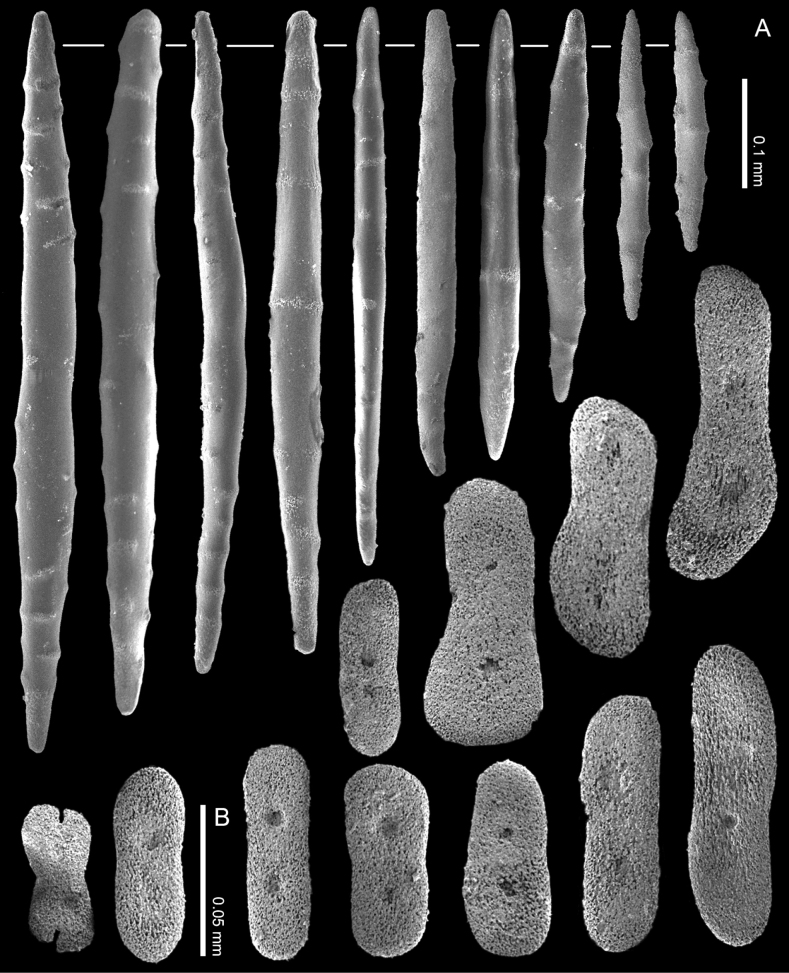
*Ofwegenumverseveldti* comb. nov., holotype SMNHTAU_Co_25554 **A** sclerites of the coenenchyme and polyp body **B** sclerites of the tentacles.

**Figure 15.**
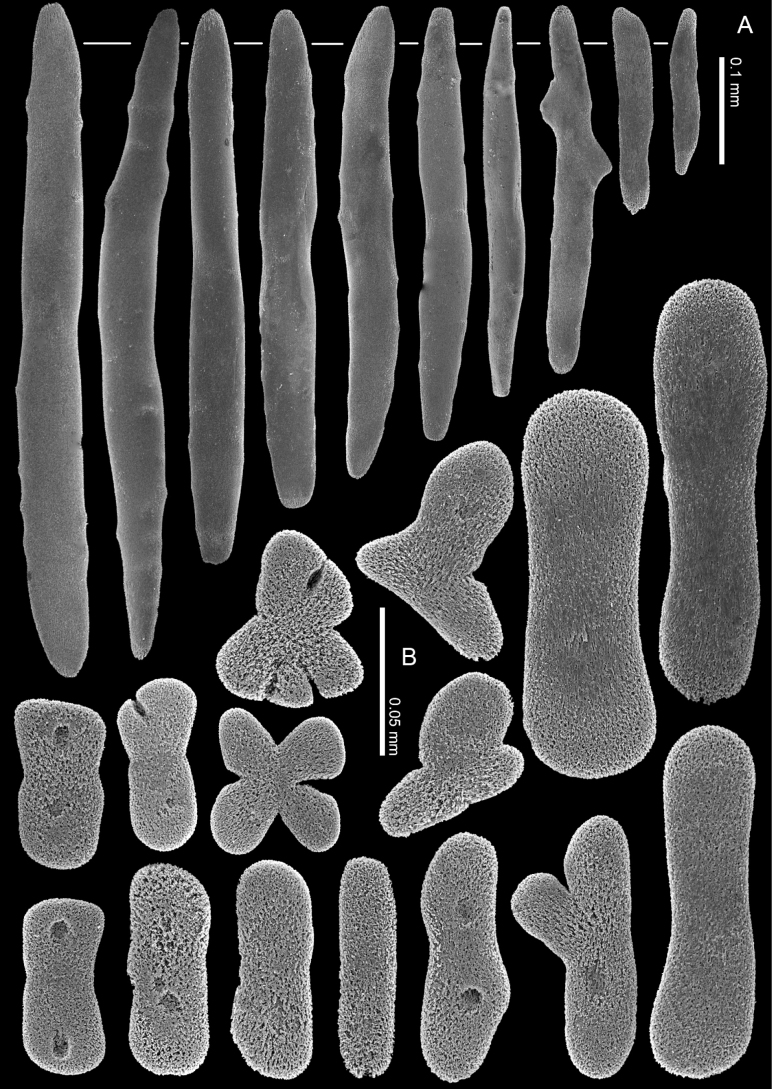
*Ofwegenumverseveldti* comb. nov., paratype, RMNH COEL. 13903 **A** sclerites of the coenenchyme and polyp body **B** sclerites of the tentacles.

Figs 1 , 3F, G , 14 , 15

##### Material examined.

***Holotype*.** Egypt • Marsa Barieka, northern Red Sea, southern tip of Sinai Peninsula; 27.7500°N, 34.2333°E; 12 m depth; 3 July 1978; coll. Y. Benayahu; SMNHTAU_Co_25554 (previously NS16770).

***Paratypes*.** Egypt • 33 colonies, same data as holotype; SMNHTAU_Co_25544 (previously NS16771) • same data as holotype; RMNH COEL. 13903.

##### Other material.

Israel• Eilat, northern Gulf of Aqaba, mesophotic reef across from the Inter University Institute for Marine Sciences (IUI); 60 m depth; 20 September 2005; coll. S. Eibinder; SMNHTAU_Co_33097.

##### Re-description

**(modified after Benayahu 1982).** The holotype is a capitate colony, 11 mm in diameter with stalk approximately 14 mm high (Fig. 3F). The contracted polyps form conical or dome-shaped mounds, and the distal ends of some tentacles can be seen protruding from them. The coenenchyme sclerites are spindles and rods up to 0.80 mm long with low, simple tubercles or areas of thickening forming concentric, raised rings (Fig. 14A). The polyp body contains similar but shorter sclerites, up to 0.45 mm long (Fig. 14A), that appear to be arranged ‘en chevron’ when the polyp is extended. The size of the sclerites decreases along the polyp body towards the base of the tentacles.

The tentacles and pinnules include numerous crosses, flattened rods (i.e., finger-biscuits) and platelets up to 0.10 mm long (Fig. 14B), arranged on the aboral side of the tentacles. Some of these sclerites have median constrictions, side notches, or depressions at one or both ends that resemble a figure-eight shape (Fig. 14B). The platelets commonly have an asymmetrical outline and are wider at both ends (Fig. 14B).

##### Colour.

In life the coenenchyme is uniquely dark blue. The expanded polyps are pale blue, with brown pinnules that reflect the presence of symbiotic algae. The ethanol-preserved colony is creamy yellow, and the tentacles are pale cream.

##### Morphological variations.

The paratype colonies and the other material vary in size; some colonies feature two separate polyparies on a common stalk (Fig. 3G). RMNH COEL. 13903 has smoother spindles and rods in both the coenenchyme and polyp body (Fig. 15A) and has fewer figure-eight platelets (Fig. 15B) compared to the holotype.

##### Remarks.

*Ofwegenumverseveldti* comb. nov. is the only species with tentacle sclerites composed mainly of asymmetrical platelets resembling a figure-eight (Figs 14B, 15B). Additionally, it has the longest spindles and rods among the congeners (Figs 14A, 15A).

The current findings correspond to the original description of *M.verseveldti* (see Benayahu 1982). The new high-quality SEM images of the sclerites (Figs 14, 15) better present the species’ diagnostic morphological characters. The tentacle sclerites reported as ‘flattened rods with tiny pits’ in the original description are referred to here as figure-eight platelets. The maximum length of these sclerites was erroneously presented by Benayahu (1982: 198, up to 0.19 mm) and is now corrected to be up to 0.10 mm (Fig. 14B). In the original description the species was described as having polyp sclerites arranged as a collaret and points, however further examination of additional material shows that is not the case. When polyps are extended the spindles and rods appear to be arranged ‘en chevron’. Benayahu (1982) also did not mention anything about the presence or absence of zooxanthellae in specimens. Re-examination of the type material confirms that *O.verseveldti* is indeed zooxanthellate.

It should be noted that despite the extensive soft coral research conducted in the Gulf of Aqaba and and other parts of the Red Sea, since the collection of the type material of *O.verseveldti* comb. nov. it has been found only once at a mesophotic depth on the Eilat reef (see above: SMNHTAU_Co_33097) and is also only infrequently observed by some professional divers in that region. This species should thus be considered as a rare soft coral in the Red Sea.

##### Distribution.

Northern Red Sea.

### ﻿Molecular results

#### ﻿DNA barcoding

Sequences for *mtMutS* (735 bp), *igr1* + *COI* (909 bp) and *28S rDNA* (800 bp) were obtained for seven specimens representing three of the four species of *Ofwegenum* plus the species from the aquarium trade (SMNHTAU_Co_38223) (Table 1). We were unable to amplify *COI* for two specimens (SMNHTAU_Co_38229, BOMAN–09174) and *mtMutS* for another (UF 15819). Only a partial fragment of *mtMutS* (450 bp) was obtained for *O.colli* sp. nov. (NTM C13089).

**Figure 16.**
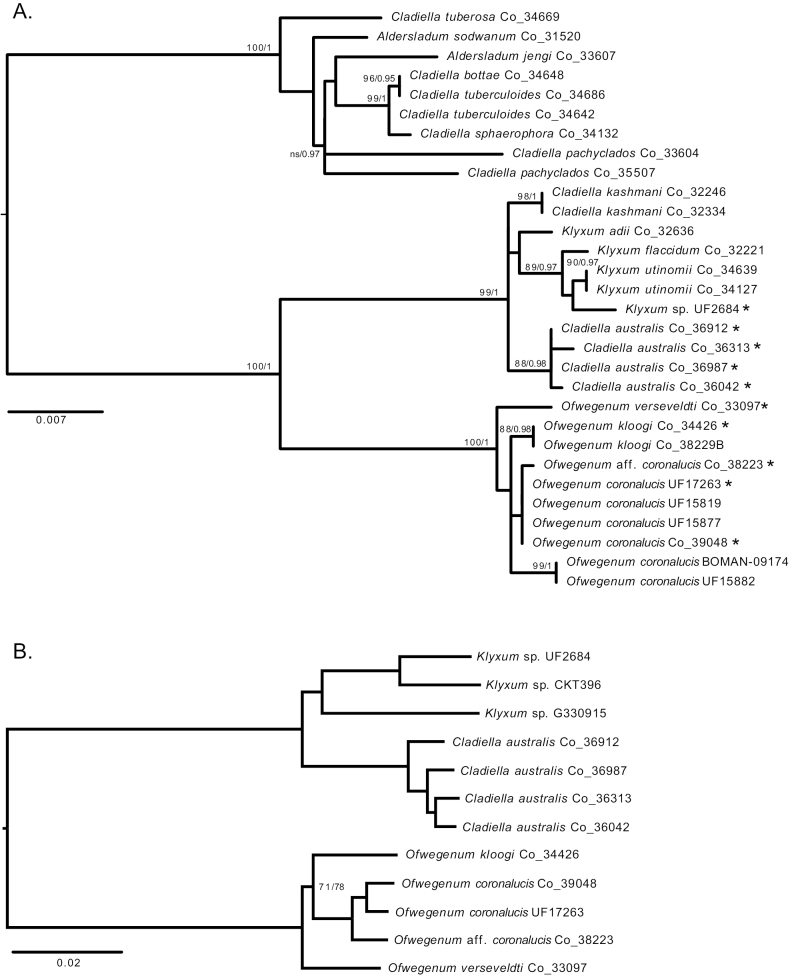
Phylogenetic relationships among species of *Ofwegenum* gen. nov. and other genera of the family Cladiellidae**A** maximum likelihood (ML) analysis of concatenated *mtMutS* and *28S rDNA* barcoding loci. Numbers at nodes: ML bootstrap percentage (10,000 ultrafast bootstrap replicates)/Bayesian posterior probability. Asterisks indicate samples that are included in analysis of conserved elements **B** maximum likelihood analysis of 1,213 conserved element loci (75% occupancy matrix). All nodes have 100% bootstrap support and SH-aLRT = 100 unless indicated. All Co_ numbers are SMNHTAU.

All phylogenetic analyses separated *Ofwegenum* gen. nov. into a well-supported clade that was sister to *Klyxum* and differed from members of that genus by mean genetic distances (uncorrected p) ranging from 1.0% (± 0.06% SD) at *COI* to 4.4% (±1.7% SD) at *28S rDNA* (Fig. 16A). Within the *Ofwegenum* clade, however, the relationships among species were poorly resolved. All *Ofwegenum* specimens had identical *mtMutS* and *COI* sequences with the exceptions of *O.verseveldti* comb. nov., which differed by a 1 bp substitution in *mtMutS*, and *O.kloogi* sp. nov. (SMNHTAU_Co_34226) which differed by a 1 bp substitution in *COI*. The partial *mtMutS* sequence for *O.colli* sp. nov. was identical to both *O.kloogi* sp. nov. and *O.coronalucis* sp. nov. At *28S rDNA*, *O.verseveldti*, *O.kloogi* and *O.coronalucis* differed from one another by genetic distances (uncorrected p) of 0.5–0.8%. The aquarium trade specimen (SMNHTAU_Co_38223) was most similar to *O.coronalucis*, differing from the holotype UF 17263 by a 1 bp substitution. There was, however, variation among individuals of *O.coronalucis*, with two specimens from Oman (BOMAN–09174, UF 15882) differing from the others by ≤ 5 bp (uncorrected p = 0.6%). Both ML and Bayesian phylogenetic analysis of the concatenated alignment of *mtMutS* with *28S rDNA* found moderate to strong support for a clade consisting of the two specimens of *O.kloogi* and a clade of the two specimens of *O.coronalucis* with divergent *28S* sequences (BOMAN–09174, UF 15882) but did not resolve the relationships among the other taxa (Fig. 16A).

#### ﻿Target-capture sequencing of conserved elements

A total of 2,509 loci (out of 3,023 targeted loci) was recovered from the assembled contigs, including the seven outgroup taxa (Table 1). The mean number of loci recovered per sample was 1,747 ± 205 SD (range: 1,297–1,977) with a mean length of 1,247 ± 92 bp SD (range: 1,121–1,395 bp). The 75% complete alignment matrix included 1,213 loci for a total length of 1,511,307 nucleotides.

The maximum likelihood analysis recovered an *Ofwegenum* clade that was strongly supported and genetically distinct from the outgroup taxa (*Klyxum* spp. and *Cladiellaaustralis*) (Fig. 16B). Within *Ofwegenum* there was strong support for a clade of *O.coronalucis* (two specimens from Oman) plus the species from the aquarium trade (SMNHTAU_Co_38223). The phylogenetic relationships among *O.verseveldti*, *O.kloogi* and *O.coronalucis*, however, remained unresolved. The single specimens of *O.verseveldti* and *O.kloogi* that were included in the analysis were equally genetically distant from *O.coronalucis*, and there was only very weak support for *O.kloogi* belonging to a clade with *O.coronalucis*.

## ﻿Discussion

The phylogenetic position of *Ofwegenum* gen. nov. as sister to the genus *Klyxum* in family Cladiellidae was well supported by both single-locus mitochondrial genes as well as the multi-locus nuclear gene analysis (Fig. 16). It shares with other members of this family polyp sclerites in the form of flattened rods and small plates with a median waist that often resemble a figure-eight. The current results demonstrate the taxonomic significance of these tentacular sclerites for species delimitation within *Ofwegenum* gen. nov. Like other Cladiellidae, only a single type of sclerite is found in the coenenchyme. The form of these sclerites—smooth spindles and rods with low protuberances that may form raised concentric rings—seems, however, to be unique within the family. Its growth form, which is encrusting or consists of small stalked polyparies in the range of a centimetre in diameter joined together in a mat, is also distinct from the predominantly lobate growth forms of other Cladiellidae. Finally, the bright blue alcohol-soluble pigments that give some *Ofwegenum* species their striking blue-green colour are unique among Cladiellidae, and rare among all octocorals. Whether or not this pigment is guaiazulene, a compound that has been found in the blue gorgonian *Guaiagorgia* and several other species (Grasshoff and Alderslade 1997) remains unknown.

*Ofwegenum* gen. nov. also shares with other genera of Cladiellidae a relatively invariant mitochondrial genome marked by little to no genetic differentiation among species at the loci commonly used for DNA barcoding (*mtMutS*, *COI*) (Benayahu et al. 2012). While *28S rDNA* exhibits greater variation among species in this clade, higher levels of intraspecific variation in that gene can also confound assessment of species boundaries (McFadden et al. 2014) as observed in *O.coronalucis* (Fig. 16A). While multi-locus methods such as the target-enrichment approach employed here generally allow species to be delimited with greater confidence (Erickson et al. 2020), our analysis of *Ofwegenum* is hampered by low sample size. Only one specimen each of *O.verseveldti* and *O.kloogi* and no *O.colli* yielded DNA of sufficient quantity and quality for library preparation. The absence of the latter species from our phylogeny and our inability to assess intraspecific genetic variation in the other two greatly limit the inferences we can make about the phylogenetic relationships and degree of genetic differentiation among species of *Ofwegenum*. Although increased sample sizes will be necessary to better resolve the species’ relationships, the apparent rarity of this genus, with each species currently known from only 1–2 locations, may hinder future attempts to increase the phylogenetic sampling.

Although it is rarely encountered in nature, *Ofwegenum* is nonetheless present in the commercial aquarium trade. We examined and sequenced a specimen (SMNHTAU_Co_38223) obtained from a supplier in the U.S. that is genetically and morphologically most similar to *O.coronalucis* (Fig. 16). The original source location of this specimen remains unknown but was thought to be Indonesia (A. Parrin, pers. comm. 12 Aug 2013), where most of the material in the U.S. commercial trade originates (Wabnitz et al. 2003). Aquarist D. Knop shared with us photos of additional specimens sourced from Indonesia (Fig. 2C–F). Whether or not any of the species we have described (or perhaps an additional species) occurs naturally in Indonesia remains unknown. Rowlett (2020) reported that a species of *Ofwegenum* has been cultured in Queensland, Australia for exportation in the aquarium trade. Whether that species might be *O.colli* sp. nov., which occurs naturally in Queensland, or the O.aff.coronalucis that is found in the aquarium trade in the U.S. also remains unknown.

## ﻿Conclusions

Here we established a new genus, *Ofwegenum* gen. nov., for *Metalcyoniumverseveldti* Benayahu, 1982. We have redescribed the type of that species and establish it as a new combination, *O.verseveldti*. In addition, we have described three new species of *Ofwegenum* from shallow-water coral reefs in the Indo-Pacific region, bringing the total number of species in the genus to four. This genus appears to be rare on coral reefs, with each species known from only a few localities, some of which have been extensively explored. The four species have distinct, non-overlapping geographical distributions, and are currently known only from the northern Red Sea (*O.verseveldti*), Arabian Sea (*O.coronalucis*), central Indian Ocean (*O.kloogi*), and northeastern Australia (*O.colli*) (Fig. 1).

## Supplementary Material

XML Treatment for Ofwegenum

XML Treatment for Ofwegenum colli

XML Treatment for Ofwegenum coronalucis

XML Treatment for Ofwegenum kloogi

XML Treatment for Ofwegenum verseveldti

## ﻿Additional information

### Author ORCIDs

Catherine S. McFadden https://orcid.org/0000-0002-8519-9762

Yehuda Benayahu https://orcid.org/0000-0002-6999-0239

Kaveh Samimi-Namin https://orcid.org/0000-0002-7744-9944

### Data availability

All of the data that support the findings of this study are available in the main text.
